# Research on the performance of modified blue coke in adsorbing hexavalent chromium

**DOI:** 10.1038/s41598-023-34381-8

**Published:** 2023-05-04

**Authors:** Hua Wang, Wencheng Wang, Guotao Zhang, Xuchun Gao

**Affiliations:** 1 College of Chemistry and Chemical Engineering, Yulin University, Chongwen Road No. 51, Yulin, 719000 Shaanxi China; 2 Shaanxi Provincial Key Laboratory of Clean Utilization of Low-Modified Coal, Yulin University, Yulin, 719000 China

**Keywords:** Environmental sciences, Hydrology, Solid Earth sciences

## Abstract

To solve the issue of hexavalent chromium (Cr(VI)) contamination in water bodies, blue coke powder (LC) was chemically changed using potassium hydroxide to create the modified material (GLC), which was then used to treat a Cr(VI)-containing wastewater solution. The differences between the modified and unmodified blue coke’s adsorption characteristics for Cr(VI) were studied, and the impact of pH, starting solution concentration, and adsorption period on the GLC's adsorption performance was investigated. The adsorption behavior of the GLC was analyzed using isothermal adsorption models, kinetic models, and adsorption thermodynamic analysis. The mechanism of Cr(VI) adsorption by the GLC was investigated using characterization techniques such as Fourier Transform Infrared Spectroscopy (FTIR), Field Emission Scanning Electron Microscope (FE-SEM), X-Ray Diffraction (XRD), and X-Ray Photoelectron Spectroscopy (XPS). With the biggest difference in removal rate at *pH* = 2, which was 2.42 times that of LC, batch adsorption experiments revealed that, under the same adsorption conditions, the GLC always performed better than LC. With a specific surface area that was three times that of LC and an average pore diameter that was 0.67 times that of LC, GLC had a more porous structure than LC. The alteration significantly increased the number of hydroxyls on the surface of GLC by altering the structural makeup of LC. The ideal pH for removing Cr(VI) was 2, and the ideal GLC adsorbent dosage was 2.0 g/L. Pseudo-second-order kinetic (PSO) model and Redlich-Peterson (RP) model can effectively describe the adsorption behavior of GLC for Cr(VI). Physical and chemical adsorption work together to remove Cr(VI) by GLC in a spontaneous, exothermic, and entropy-increasing process, with oxidation–reduction processes playing a key role. GLC is a potent adsorbent that can be used to remove Cr(VI) from aqueous solutions.

## Introduction

In recent years, with the rapid development of industry, agriculture, and urbanization, environmental protection issues have aroused wide public concern^1^. Chromium, a significant industrial material, is widely used in processes such as paper making, electroplating, dye manufacturing, leather tanning, and painting^2,3^. If large amounts of chromium-containing waste are released into the environment, serious environmental pollution may occur. Chromium mainly exists in nature in the form of Cr(VI) and Cr(III)^4^. Cr(VI), such as Cr_2_O_7_^2−^ and HCr_2_O_7_^−^, is highly toxic and mobile, while Cr(III) has low solubility in water, low flow mobility, and biorelease^5,6^. The main target of chromium pollution management is Cr(VI), which tends to accumulate in the human body during the metabolic process and can cause a series of health problems, such as skin irritation and lung cancer^7,8^. To solve the problems caused by Cr(VI) pollution, researchers have developed various methods to treat Cr(VI), including chemical reduction, adsorption, bioremediation, electrocoagulation, etc.^9^. Among them, adsorption has attracted much attention due to its advantages such as economy, high efficiency, and feasibility^10^. Carbon materials as adsorbents have been widely used to remove heavy metals due to their high surface area, abundant functional groups, and good chemical stability^5^.

Considering sustainability and conservation, a cost-effective carbon adsorbent, like accessible industrial wastes, is a practical choice for wastewater treatment^11^. Blue coke, a type of semi-coke produced by pyrolyzing non-caking or weakly caking coal with a highly volatile component at low temperatures^12^, is the product of the clean utilization of coal^12^. It has been widely used as a reductant for the manufacturing of ferroalloys and calcium carbide, as a jet feedstock for blast furnaces, and as a clean fuel for both industrial and public use^13^. It is usually granular coke with a particle size larger than 6 mm^12^. Semi-coke of powder is a byproduct of the production and processing of blue coke, which is either discarded or sold cheaply^14^. However, due to its low price, high caloric value, and capacity to be used as a fuel for a coke furnace to recover energy and remove the absorbed resistant pollutants, it has recently become a popular adsorbent^15^. The production cost of activated carbon (AC) can be reduced by roughly 500 CNY t^−1^ by adding 5% powder blue coke in the process^16^. Nevertheless, raw powder coke is not often used due to its limited surface area and character as a non-selective adsorbent and non-reactive chemical^17^. As a potent adsorbent, it must be modified before use. It was found that powder blue coke can have good adsorption performance and load function when using a multi-aperture and large-ratio surface^18^. However, the features of the objective adsorbate might affect the modifying method for blue coke powder^19^. By providing sufficient functional groups and a high specific surface area, Cr(VI) can be eliminated from wastewater.

However, there is limited research on the process and efficiency of blue coke in removing Cr(VI). The study aims to investigate the potential of using powder blue coke-based adsorbents to treat wastewater containing chromium, thereby transforming the waste or inexpensive powder coke into a valuable product, following the waste-to-wealth principle. A useful adsorbent was made by modifying the surface structure of powder blue coke for the treatment of Cr(VI). The powder blue coke was treated with potassium hydroxide in a nitrogen atmosphere to increase the amount of functional groups and surface area. The main goals of the research presented in this paper are: (1) examine the effectiveness of blue coke in removing Cr(VI) from water through sorption experiments; (2) evaluate the influence of various factors, such as the dose of blue coke, *pH*, initial Cr(VI) concentration, temperature, and contact time on its effectiveness; and (3) determine the mechanisms of Cr(VI) removal by blue coke. This research can serve as a useful guide for the industrial production of carbon material adsorbents based on powder coke. Understanding the theoretical and practical significance of waste recovery will aid the coking industry in establishing a recycling economy.

## Experimental methods

### Materials and reagents

The raw material of powder blue coke was obtained from a coking plant in Yulin, Shaanxi. The reagents used in the experiments were purchased from Tianjin Zhiyuan Chemical Reagent Co. The reagents were all analytically pure and had not undergone any processing.

### Material preparation

The blue coke from the coking plant was crushed and passed through a 100 mesh sieve. Then it was washed several times (not less than three times) with deionized water. The cleaned blue coke was dried to constant weight in a blast dryer (80 °C). The prepared sample was labeled as LC. 400 mL of sodium hydroxide solution at a concentration of 5% (w/v) was added to a conical flask containing 10 g of the prepared LC sample. The mixture was then agitated magnetically for 6 h and then allowed to stand overnight before the solid and liquid components were separated. The filtered samples were placed in a tube furnace and heated to 800 °C at a rate of 10 °C /min while being exposed to an environment of N_2_ flowing at a rate of 100 mL/min. After holding for 1 h, the samples were then allowed to cool. Following cooling, the samples were periodically washed in deionized water until the pH value remained constant, after which they were dried in a blast drying oven at a constant temperature of 80 °C. The obtained samples were labeled as GLC, respectively.

### Adsorption experimental method

Hexavalent chromium mother liquor configuration method: Dissolve 2.829 g K_2_Cr_2_O_7_ in 1 L deionized water. It was diluted using a certain number of times in accordance with the needs of the experiment. The concentration of Cr(VI) was determined by the spectrophotometric method of diphenylcarbonyl dihydrazine. GLC of dosages ranging from 50 to 250 mg was added to a 100 mL solution containing 50 mg/L of Cr(VI). Following a 24-h incubation period at 298 K and constant oscillation speed, the Cr(VI) content was determined. The effect of pH (ranging from 2 to 10) on the removal of Cr(VI) was investigated using 50 mL of a solution containing 50 mg/L of Cr(VI) and 100 mg of the sample. Kinetic experiments were conducted by adding 1 g of GLC to a 500 mL solution containing 50 mg/L of Cr(VI) at pH 2, with samples taken at intervals during 0 to 24 h. A total of 7 supernatant samples, each with a volume of no more than 1 mL, were taken. To assess the adsorption isotherms and thermodynamics, 100 mg of GLC was added to a 50 mL solution containing Cr(VI) concentrations ranging from 10 to 400 mg/L at pH 2 and temperatures of 298 K, 308 K, and 400 K. The GLC was regenerated after it had been saturated with adsorbed Cr(VI) using a 0.05 mol/L sodium hydroxide solution. The GLC’s reusability was evaluated using the repeated adsorption and desorption process. The detailed operation steps are as follows. In an acidic environment (pH 2) at 298 K, 20 mg of adsorbent was added to 20 mL of a solution containing 50 mg/L of Cr(VI), and the mixture was left to react for 6 h at 120 rpm. After that, the adsorbent was separated by centrifugation and washed with 0.05 mol/L NaOH solution. Then, it was soaked for 6 h in 20 mL 0.05 mol/L NaOH at 120 rpm, and the Cr(VI) was removed. Thereafter, the adsorbent was rinsed with ultrapure water until the eluent pH was neutral. The regenerated adsorbent was isolated. Afterward, this operation was repeated five times.

The adsorption amount *Q*_*t*_ (mg/g) and removal rate *R* of Cr(VI) by the materials were calculated using the following equations, respectively.

1 $${Q}_{t}=\frac{({C}_{0}-{C}_{t})\times V}{m},$$

2 $$ R = \frac{{C_{0} - C_{t} }}{{C_{0} }} \times 100\% , $$

where: *C*_*0*_ (mg/L) is the initial Cr(VI) concentration in the solution; *C*_*t*_ (mg/L) is the Cr(VI) concentration at time t in the solution; *V* (L) is the solution volume; *m* (g) is the amount of material added.

### Sample characterization

FE-SEM (SIGMA 300, Germany) was used to observe the surface morphology and internal microstructure of the materials. The pore structure parameters of GLC and LC were measured and calculated using a fully automated comparative area analyzer (ASAP 260, Micromeritics Instrument Corp, USA). The material surface functional group information was identified by FTIR technique (TENSOR 27, Bruker, Germany) in the range of 400 ~ 4000 cm^−1^. The surface characterization of GLC was performed using XPS (Thermo Scientific K-Alpha, USA). The crystal structure data of the materials were obtained from XRD (D8-Advance, Germany). Cr(VI) concentrations were determined by UV–Vis spectrophotometer (722 s, Shanghai Jing Hua, China). Total chromium determined by atomic absorption spectrophotometer (AA-6300, Shimadzu, Japan). The pH values were determined by an acidity meter (PHS-3C, Shanghai Lei Magnetic, China). In this study, the pH drift method^20^ was used to estimate the point of zero electric charges (pH_pzc_).

### Adsorption kinetics

Four kinetic models were used to examine how quickly the adsorption of Cr(VI) by GLC occurs.

PFO^21^:

3 $${Q}_{t}={Q}_{e}\left(1-{e}^{-{k}_{1}t}\right),$$

PSO^21^:

4 $${Q}_{t}=\frac{{{k}_{2}Q}_{e}^{2}t}{1+{k}_{2}{Q}_{e}t},$$

Elovich model^22^:

5 $${Q}_{t}=\frac{1}{\beta }\mathrm{ln}\left(\beta \alpha t+1\right),$$

WM^23^:

6 $${Q}_{t}= {k}_{i}{t}^{1/2}+C,$$

where: *t* (h) denotes the time of adsorption; *Q*_e_ (mg/g) and *Q*_t_ (mg/g) are the equilibrium adsorption amount and the adsorption amount at time t, respectively; *k*_1_ (min^–1^), *k*_2_ (g·mg^–1^ min^–1^) and *k*_i_ (mg· g^–1^ h^–1/2^) are the rate constants calculated by PFO, PSO, and WM, respectively; *α* (mg·g^–1^·h^–1^) and *β* (g·mg^–1^) are the adsorption rate constants and desorption constants calculated by the Elovich model, respectively; *C* (mg·g^–1^) is a constant related to the thickness of the boundary layer, and the higher value of *C* corresponds to the greater effect of the limiting boundary layer^23^.

### Adsorption isotherm analysis

The adsorption isotherm of Cr(VI) on GLC was fitted using the Langmuir model, the Freundlich model, and the RP model. Equation (7) is the Langmuir model, which describes the adsorption of homogeneous single-molecular layers^20^. Equation (8) is the Freundlich model, which describes the adsorption of non-homogeneous multi-molecular layers^24^. Equation (9) is the RP model, which describes both homogeneous and non-homogeneous surfaces^25^.

Langmuir model^22^:

7 $${\text{Q}}_{\text{e}}= \text{ } \frac{{\text{Q}}_{\text{max}}{{\text{K}}}_{1}{\text{C}}_{\text{e}}}{\text{1} + {\text{K}}_{1}{\text{C}}_{\text{e}}},$$

Freundlich model^24^:

8 $${\text{Q}}_{\text{e}}= \text{ } {\text{K}}_{2}{{\text{C}}}_{\text{e}}^{\text{1/n}},$$

RP isothermal adsorption model^25^:

9 $$ Q_{e} = \frac{{K_{3} C_{e} }}{{1 + \alpha C_{e}^{\beta } }}, $$

where *K*_1_(L·mg^–1^), *K*_*2*_(mg^1–1/n^·L^1/n^·g^–1^), and *K*_*3*_(L·g^–1^) are the constants corresponding to the three isothermal adsorption models associated with the adsorption energy, respectively; *Q*_*max*_(mg/g) is the maximum adsorption amount fitted by the Langmuir model; *n* is the constant of the Freundlich model associated with the relative adsorption strength; *α* and *β*(L^β^·mg^–β^) are the constants corresponding to the RP isothermal.

### Thermodynamic analysis of adsorption

The thermodynamic adsorption parameters are calculated as shown below^8,26^:

10 $${K}^{\theta }={{C}^{\theta }\times M}_{mol}\times {K}_{3},$$

11 $${\Delta G}^{0}=-RTln\left({K}^{\theta }\right),$$

12 $$ln\left({K}^{\theta }\right)=\frac{{\Delta S}^{\theta }}{R}-\frac{{\Delta H}^{\theta }}{RT},$$

where ${K}^{\theta }$ is the standard equilibrium constant. The value of ${K}^{\theta }$ was calculated using the method proposed by Tao Chen et al^26^. The value ${C}^{\theta }$ is taken as the IUPIC recommended value of 1 mol/L. ${M}_{mol} (\mathrm{g}/\mathrm{mol})$ is the molar mass of chromium. ${K}_{3}$ (L/g) is the constant of the RP model related to the adsorption energy. *R* (J·mol^−1^·K^−1^) is the ideal gas constant. The values of $\Delta {H}^{\theta }$ and $\Delta {S}^{\theta }$ were calculated by linear fitting.

## Results and discussion

### Properties of the samples

#### Surface morphology

**Figure 1:**
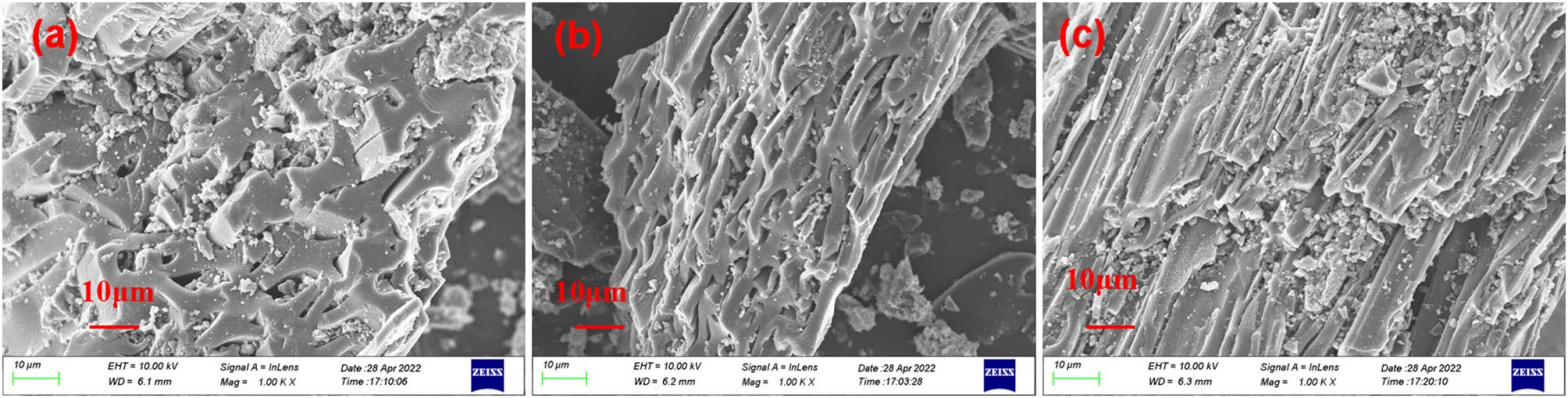
FE-SEM images: (**a**) LC; (**b**) GLC; (**c**) GLC-Cr.

FE-SEM was used to examine the microscopic morphology of LC, GLC, and Cr-adsorbed GLC (GLC-Cr). As can be seen in Fig. 1a, the surface of LC has a distinct pore size distribution structure, although it is mostly covered in impurities, which may be the ash that LC formed during the formation process. In comparison to LC, GLC has a surface that is more uneven and fluffy, which reveals a more advanced pore distribution (Fig. 1b). It reveals that the initially filled pore channels in LC were obviously emptied after modification by potassium hydroxide. Most of the pores were filled when Cr(VI) was adsorbed by GLC (Fig. 1c). Additionally, it was observed that GLC had a large number of fine, crumbs-like materials adsorbed on its surface (Fig. 1c). This implies that precipitates developed as Cr(VI) were being eliminated.

#### Specific surface area, pore size, and pore volume analysis

**Figure 2:**
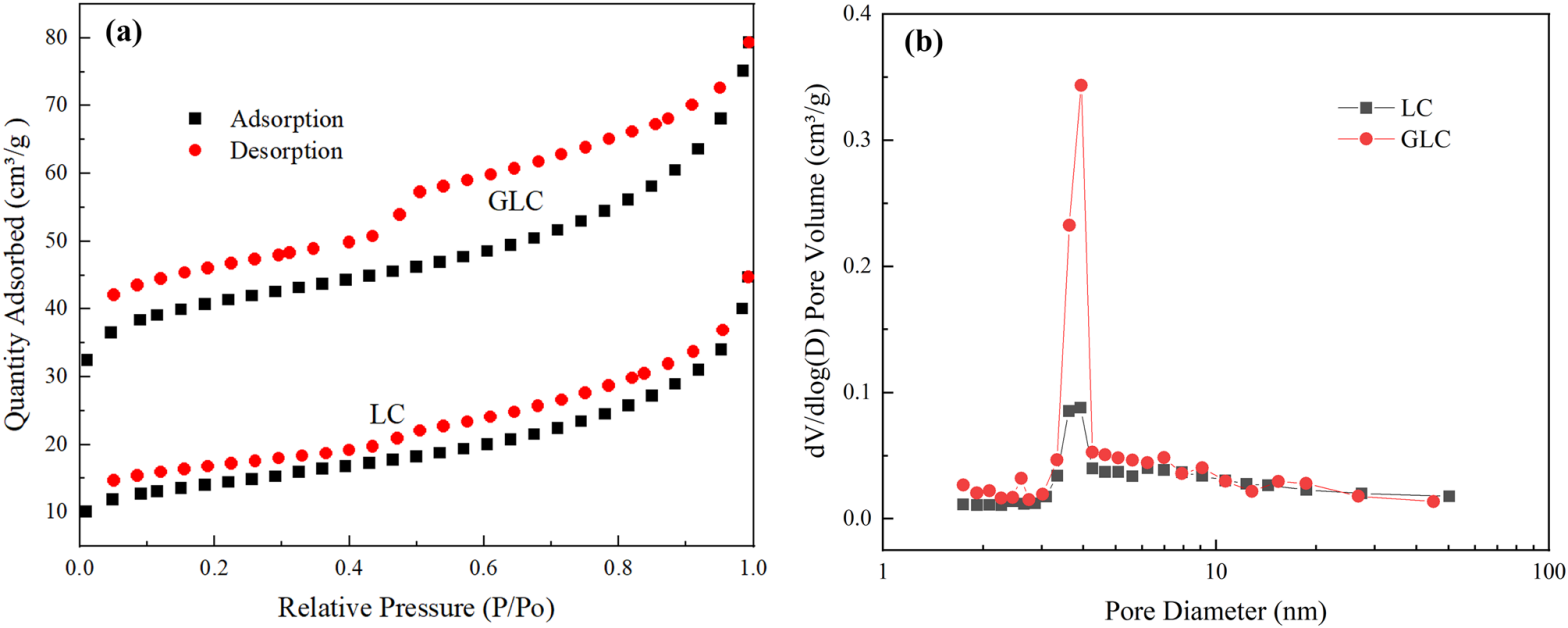
(**a**) The N_2_ adsorption and desorption curves of the samples; (**b**) The pore size distribution characteristics of the samples.

The pore structure and specific surface area of the adsorbent significantly influence the capacity for removing Cr(VI). The N_2_ adsorption and desorption curves of GLC and LC were shown in Fig. 2a. It can be seen that the adsorption and desorption curves of the two materials partially overlapped, and a definite hysteresis loop appeared where *p/p*_*0*_ was greater than 0.5, indicating that both materials had mesoporous structures^27^. At *p/p*_*0*_ greater than 0.9, the N_2_ adsorption–desorption curve continued to grow significantly, demonstrating that both materials also include some macroporous^28^. The modified GLC has a more abundant porosity structure than the unmodified LC, as seen by the N_2_ adsorption and desorption curves of GLC being above the LC’s curves.

The pore size distribution characteristics of the GLC and LC are depicted in Fig. 2b. It can be seen that the pore size distribution of GLC is essentially the same as that of LC, with mesoporous materials primarily located in the 2–10 nm range, which is consistent with the results of the N_2_ adsorption and desorption curve. However, GLC's pore abundance is substantially better than LC's at the same pore size, showing that the addition of a potassium hydroxide activator can encourage the formation and growth of the pore structure. The details of the pore structure parameters are shown in Table 1. As can be observed, GLC has a three-fold larger specific surface area than LC, as well as higher micropore areas, total pore volumes, and micropore volumes than LC. However, compared to before the alteration, the average pore size is now smaller. It demonstrates that pore channel expansion and the stimulation of the formation of microporous and mesoporous structures are both benefited by potassium hydroxide activation.

**Table 1:** Structural parameters of the samples.

Sample	S_BET_ m^2^/g	S_Micro_ m^2^/g	V_Total_ cm^3^/g	V_Micro_ cm^3^/g	D_p_ nm
LC	51.099	21.324	0.0556	0.00894	4.351
GLC	153.742	106.104	0.112	0.0424	2.917

#### XRD analysis

**Figure 3:**
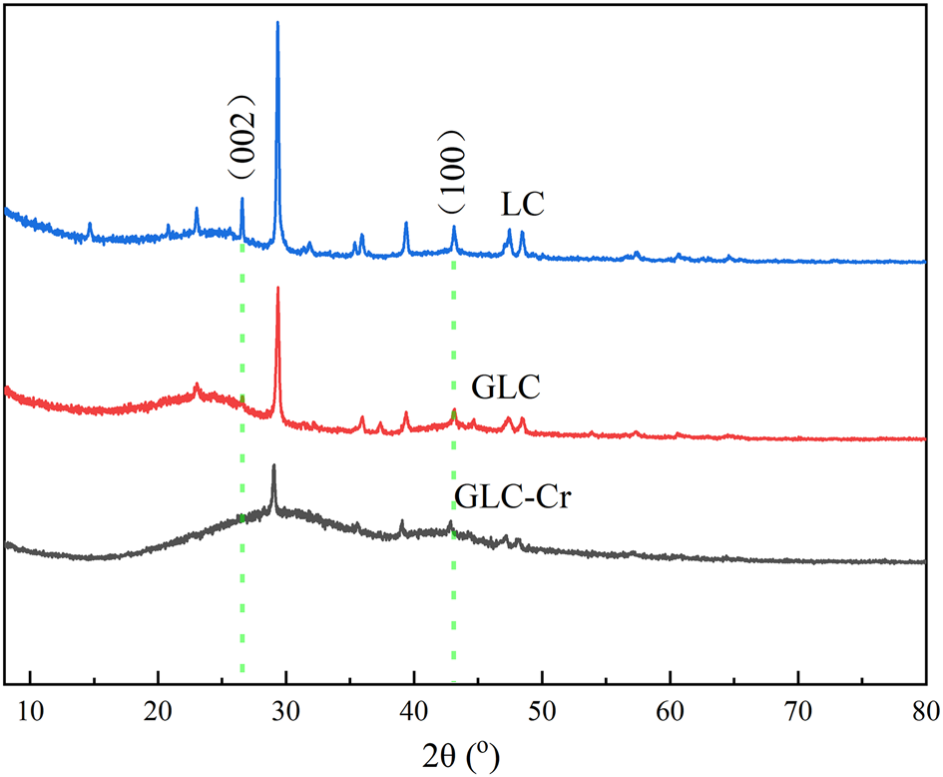
XRD patterns of the samples.

The XRD spectra of LC, GLC, and GLC-Cr are shown in Fig. 3. The distinct peaks of LC and GLC are comparable, as can be observed, while those of GLC is noticeably weaker when compared to some inorganic salt components. This might be a result of the activation process of acid and water washing. Additionally, the typical absorption peaks of carbon materials (near 2θ = 26$^\circ$ and 43$^\circ$) were seen in the LC and GLC spectra. They correspond to the (002) and (100) microcrystalline diffraction peaks of graphite microcrystals^29^. However, the GLC diffraction peaks’ strength was noticeably lower than that of the LC, proving that the graphitization was weaker as a result of the activation. The characteristic diffraction peaks of graphite microcrystals of GLC-Cr became much weaker, proving that the process of Cr(VI) adsorption disrupted the structural makeup of the GLC surface.

#### FTIR analysis

**Figure 4:**
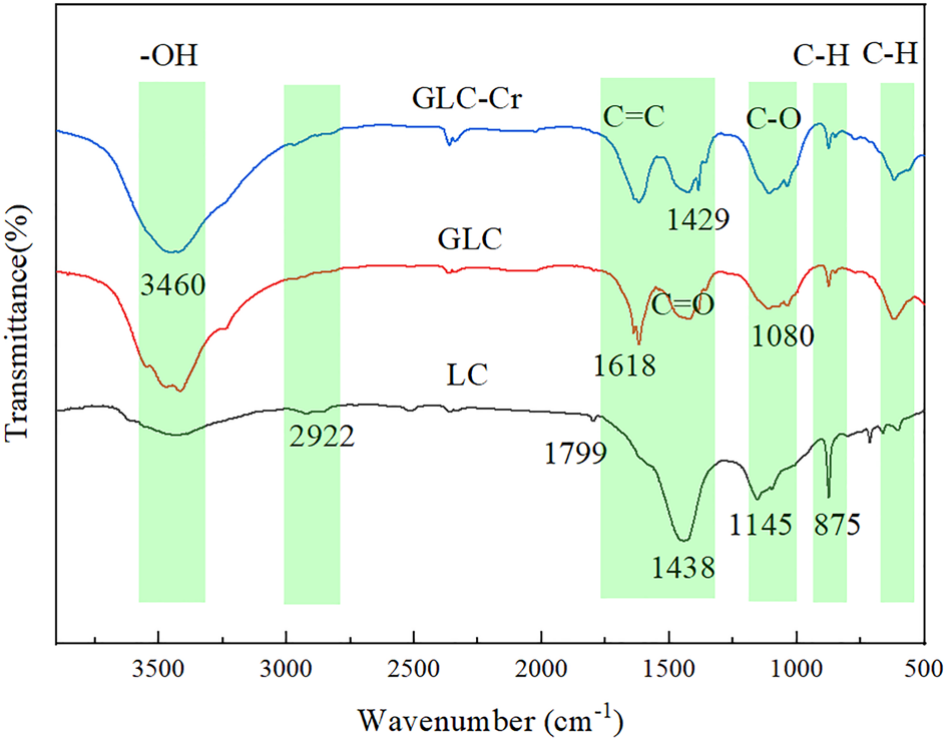
FTIR spectra of the samples.

FTIR was used to analyze the functional groups on the surfaces of LC, GLC, and GLC-Cr (Fig. 4). As can be seen, the peak of the infrared spectrum of GLC is markedly different from that of LC, indicating that the activation of potassium hydroxide changed both the structure and number of functional groups on the material's surface. The peak near 3420 cm^–1^ was caused by the stretching vibration of O–H^30^. The peak intensity of GLC at this position was bigger than LC, showing that activation improved the quantity of hydroxyl functional groups on the surface of the material. The peaks in the range of 2800–3000 cm^–1^ are caused by symmetric or asymmetric stretching vibrations of –CH_2_ or –CH_3_^31^. GLC at this position had a lower peak intensity than LC, indicating that the surface's aliphatic structure was destroyed by activation. The peaks in the range of 1720–1320 cm^–1^ are caused by C=C skeleton vibrations or C=O stretching vibrations^32–34^. The peaks of C=C and C=O functional groups on the surface of LC overlapped to form a broad absorption peak^35^. However, the peaks on the surface of GLC were distinct, indicating that the activation led to a reorganization of the functional groups on the material’s surface^36–38^. The peak near 875 cm^−1^ is caused by the stretching vibration of aromatic –CH^31^. The peak intensity of GLC at this site is obviously lower than LC, suggesting that the modification may have partially destroyed the aromatic structure, which is consistent with the results of the XRD investigation above^37,39^. It is clear by comparing the peak changes before and after adsorption that the peak of –OH became wider and weaker; the peak intensity of C=O showed a slight change, and the peak intensity of C–O increased. It is assumed that the oxygen-containing functional groups on the surface of GLC are involved in the adsorption reaction^40^.

### The adsorption performance of GLC

#### The effect of GLC dosage

**Figure 5:**
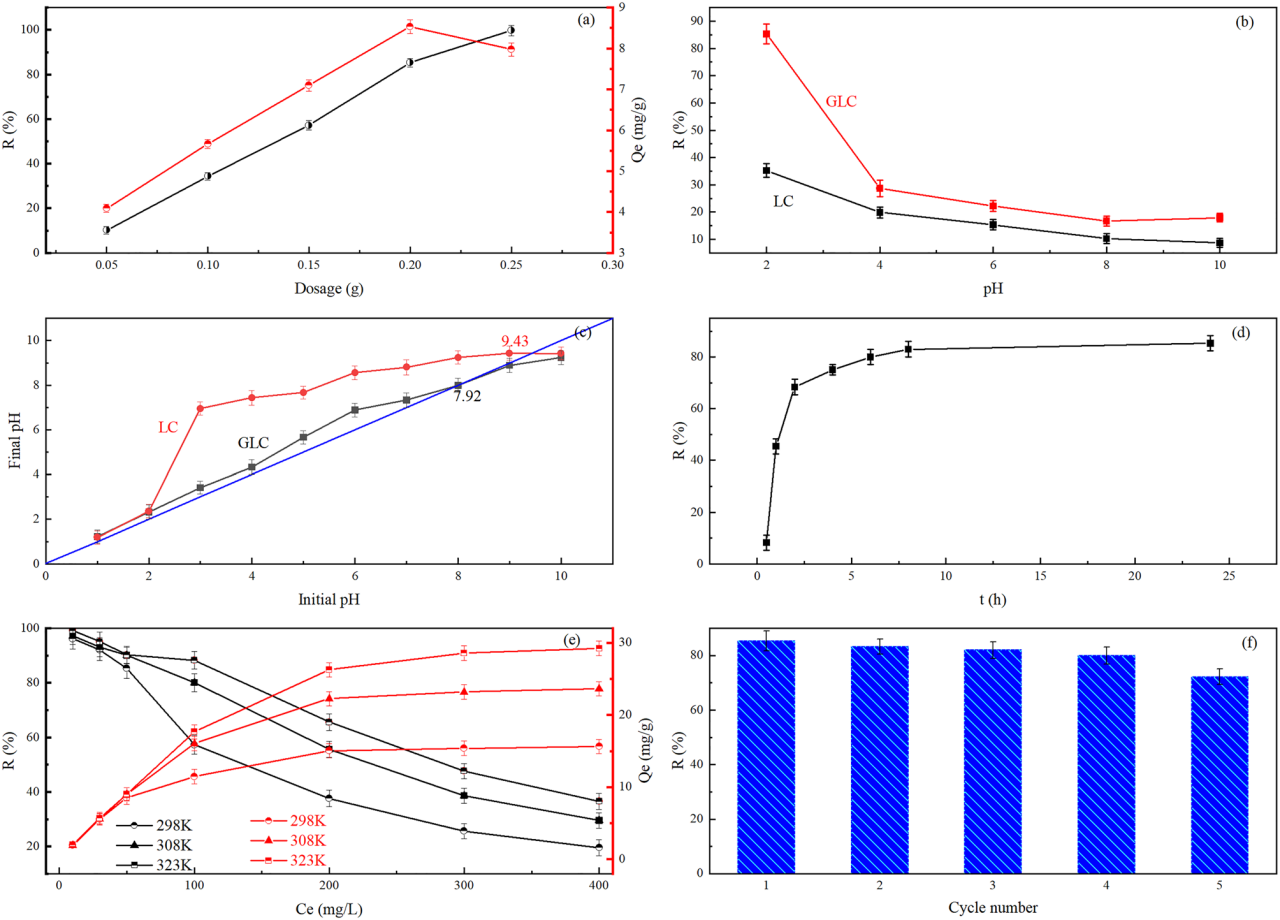
(**a**) Effect of the samples’ dosage on the Cr(VI) removal capacity and the adsorption amount of Cr(VI) by GLC (**b**) Effect of the solution pH on the Cr(VI) removal capacity; (**c**) Effect of the initial pH on the final pH; (**d**) Effect of the adsorption time on the the Cr(VI) removal capacity; (**e**) Effect of the initial Cr(VI) concentration on the Cr(VI) removal capacity and the adsorption amount of Cr(VI) by GLC; (**f**) Effect of number of regeneration on the Cr(VI) removal capacity. All error bars represent the standard error of the mean for each data point.

The effect of GLC dosage on the adsorption and elimination of Cr(VI) was depicted in Fig. 5a. It can be seen that the removal rate of Cr(VI) increased continuously when the dosage was increased. However, with the highest removal rate of 99.69%, the rise of the removal rate exhibited a tendency to first increase and then weaken. It implies that the quantity of active sites on the adsorbent's surface increases as its dosage does^41,42^. Additionally, it was demonstrated that the unit adsorbed amount and Cr(VI) removal rate differed dramatically with increasing GLC dosage. The amount of adsorption initially increased and then decreased. At a dosage of 2.0 g/L, it peaked at 8.532 mg/g. The reasons for the improved removal effectiveness were the increased total surface functional groups of the adsorbent and the increased effective contact area, which supplied more active sites. In contrast, the decrease in the usage of active sites at high dosages was the cause of the decrease in the unit adsorption capacity of Cr(VI). This is a result of larger adsorbent dosages causing agglomeration of adsorbent particles^43^ or the adsorption sites remaining unsaturated and the active sites remaining underutilized^44^. This is in line with the findings showing that the adsorption capacity of Cr(VI) dropped from 14.5 to 4.0 mg/g as the dosage of adsorbent was raised from 0.25 to 5 g at a constant starting concentration of Cr(VI) in a 50 mL solution^45^. The findings demonstrated that while the removal rate of Cr(VI) can be increased by merely raising the dosage of adsorbent, doing so will result in adsorbent waste. In light of the economic and effect evaluation, 2.0 g/L was determined to be the ideal dosage of GLC.

#### The effect of pH

The pH of the solution affects both the degree of protonation of the functional groups and the charge on the surface of the adsorbent^46,20^. The present state of chromium is also influenced by the pH in the solution^47^. The occurrence mode of Cr(VI) in the solution has a direct impact on the removal efficiency of Cr(VI) by the adsorbent^48^. The removal rate of Cr(VI) for modified GLC and unmodified LC is affected by pH, as shown in Fig. 5b. The removal rate of Cr(VI) by both materials showed a strong decreasing trend with rising pH, especially in the pH range of 2–4, where the removal rates of GLC and LC fell from 85.32 to 26.68% and from 35.23 to 19.88%, respectively.

The present state of Cr(VI) in solutions with various pH values^20^: When the pH is less than 1, the nonionic H_2_CrO_4_ gradually replaced the ionic Cr(VI) presence^49^; When the pH was between 1 and 8, it was primarily present as HCrO_4_^-^ and Cr_2_O_7_^2–^^50^; when the pH was more than 8, the primary form was CrO_4_^2–^^51^. In this research pH range, Cr(VI) is primarily present as the anions Cr_2_O_7_^2–^, CrO_4_^2–^, and HCrO_4_^–^. The results of the zero electric point measurements for the two materials are shown in Fig. 5c, with 7.92 for GLC and 9.43 for LC. The sample’s surface was protonated and positively charged when the pH of the solution was lower than the sample’s zero electric point^52,53^ . The elimination of Cr(VI) below the sample's zero charge point was influenced by the electrostatic attraction between the anion Cr(VI) and the positive charge on the sample surface. There is the more positive charge on the adsorbent sample surface, the stronger the affinity for the anion Cr^54^. When the pH is higher than 7, there are more OH^–^ ions in the solution, which compete with Cr(VI) anion^55^. In addition, compared to HCrO_4_^–^, CrO_4_^2–^ has a high adsorption energy^56^. This also wasn’t a benefit for removing Cr(VI).

The researchers also discovered that a substantial amount of H^+^ ions are consumed by the adsorbent during the removal of Cr(VI), and that consumption tends to decline as *pH* increases^20^. When a significant number of H^+^ ions were present, Cr(VI) was changed to Cr(III). The specific reaction equation is as follows^57^:

13 $${Cr}_{2}{O}_{7}^{2-}+14{H}^{+}+6{e}^{-}\to 2{Cr}^{3+}+7{H}_{2}O,$$

14 $$H{Cr}_{2}{O}_{7}^{-}+7{H}^{+}+3{e}^{-}\to {Cr}^{3+}+4{H}_{2}O,$$

15 $${Cr}_{2}{O}_{7}^{2-}+8{H}^{+}+3{e}^{-}\to {Cr}^{3+}+4{H}_{2}O.$$

In strong acidic solution (pH < 1), the removal rate of total chromium increased while the removal rate of Cr(VI) declined, and total chromium removal rates were always lower than Cr(VI)^57^. This reveals that the conversion of Cr(VI) to trivalent chromium benefits with an increase in the acidity of the solution, and a part of trivalent chromium ions occurred in the solution. In this experiment, with the increase of *pH*, the removal rate of Cr(VI) decreased significantly. This phenomenon was caused by the redox and the electrostatic attraction. The effect of redox is stronger than electrostatic attraction in strongly acidic solutions. Based on the experimental results, *pH* 2 was selected as the subsequent experimental condition to explore the removal performance and mechanism of Cr(VI) by GLC.

#### Regeneration results

The regeneration of the adsorbent is its most crucial part since it allows for its reutilization and long-term viability. NaOH solution was used in GLC recovery testing because it can effectively desorb Cr by rupturing the link between the adsorbent and the Cr anion^58^. The removal efficiency of Cr(VI) by GLC in each cycle was 85.32%, 83.31%, 82.00%, 80.01% and 72.23%, as shown in Fig. 5f. After five cycles, the removal capacity dropped from 85.32 to 72.23%. This suggests that the majority of active sites can be successfully restored. Although it decreased slightly, the removal efficiency of Cr(VI) was still very satisfactory. GLC can be regarded as one of the most effective and promising Cr(VI) adsorbents due to its excellent reusability.

### Adsorption kinetics analysis

The effect of adsorption time on the removal rate and adsorption amount of Cr(VI) are shown in Fig. 5d. In the initial stage (0–4 h), the removal rate of Cr(VI) by GLC increased dramatically, showing that the majority of the adsorption took place on the adsorbent's outer surface^59^. At this stage, physical adsorption plays an important role^60^. The increasing trend of removal rate decreased significantly (4–10 h) with the adsorption time, indicating that a sizeable portion of Cr(VI) would enter the pore channel and adsorb on the inner surface active sites with the decrease of the outer surface active sites. Cr(VI) diffuses slowly through the pore channel, causing the adsorption on the inner surface to have a higher adsorption resistance than that on the outer surface and to take longer^61^. As the adsorption time increases, the active sites on the outer surface are depleted, and the majority of the adsorption occurs on the inner surface. As a result, the removal rate increases less quickly, and the adsorption eventually reaches equilibrium when the active sites on the inner surface are also depleted.

The characteristics of the adsorption process can be obtained by analyzing the effect of contact time on the adsorption amount. Time has a comparable effect on the adsorption amount of Cr(VI) as it does on the elimination rate of Cr(VI). The quick adsorption was completed within 4 h. At 4 h, there was an adsorption amount of 6.632 mg/g, which is 77.73% of the maximum value. During the rapid adsorption phase, the adsorption amount increased significantly with time due to the presence of a large number of active adsorption sites on GLC. With the decrease in the number of adsorption sites, the sample entered the slow adsorption phase, where the adsorption rate slowed down and gradually approached equilibrium^62^.

Kinetic experiments are needed to determine the removal rate of Cr(VI) by GLC. PFO kinetic model can be used to explain the adsorption process as physical adsorption with single molecular layer adsorption, where the diffusion step controls the adsorption rate^23^. PSO kinetic model depicts a chemical adsorption process involving the sharing or transfer of electron pairs between the adsorbent and the adsorbate^63^. The adsorption rate of Cr(VI), in this instance, is governed by chemical interactions rather than the velocity of material transit. Elovich model describes the chemisorption process on the non-homogeneous surface of the adsorbent^23,64^.

**Figure 6:**
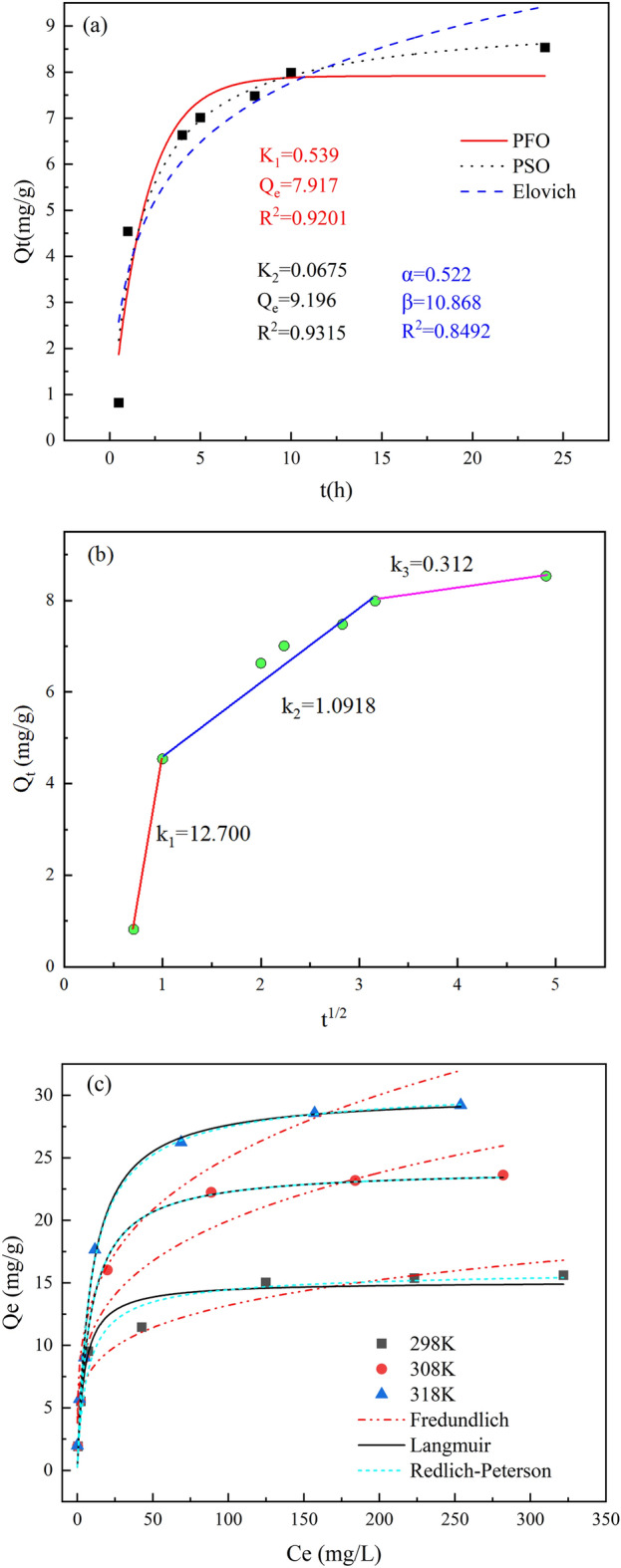
(**a**) Kinetic fitting results by PFO, PSO and Elovich model; (**b**) Intra-particle diffsion model; (**c**) Adsorption isotherm fitting results.

The adsorption behavior of GLC was explored by using these three kinetic models. The fitted curves and parameters are shown in Fig. 6a. As can be seen, compared with the fitted PFO and Elovich models, PSO fits better and has the highest R^2^ value (0.9315). This demonstrates that the adsorption process of Cr(VI) by GLC is controlled more by chemisorption than by the material transit step, such as surface complexation and precipitation, etc. Therefore, the removal of Cr(VI) by GLC is mainly based on the control of surface chemical reactions^65^.

The Weber-Morris model was employed to investigate the adsorption rate control mechanisms more. The fit's result is displayed in Fig. 6b. As can be observed, none of the trend lines that was fitted by the data passed through the origin, suggesting that a multi-step process controls the adsorption^66^. The Weber-Morris model distinguishes between the three stages of the adsorption process: liquid film diffusion, intraparticle diffusion, and adsorption equilibrium. The three stages’ rate constants are in the following order: *k*_*1*_ > *k*_*2*_ > *k*_*3*_, indicating that the liquid film diffusion and intraparticle diffusion are the controlling steps of the adsorption process. The short duration of the first stage and the significant increase in the amount of adsorption indicate that the many functional groups on the outer surface of GLC appear to play a particularly significant role in the adsorption process^67^. In this stage, Cr(VI) can be rapidly trapped at the material surface, implying that surface diffusion is the rate-controlling factor^68^.

### Adsorption isotherm and adsorption thermodynamic analysis

#### Effect of initial concentration

The effect of initial concentration on the removal of Cr(VI) by GLC is shown in Fig. 5e. As can be observed, the removal rate of Cr(VI) tends to drop as the initial concentration increases, and as the initial concentration increases, the declining tendency gradually reduces. When the starting concentration was less than or equal to 30 mg/L at any of the three temperatures, the clearance rate was better than 90%; however, even at 318 K, it was still less than 50% when the concentration reached 300 mg/L. In contrast to the removal rate of Cr(VI), the amount of adsorption increased as the solution's initial concentration rose. This was caused by the concentration gradient increasing for Cr(VI) between the liquid phase main body and the surface of GLC^69^. More active spots on the surface of GLC were occupied in proportion to the force of mass transfer for Cr(VI)^70^. On the GLC surface, there are only a limited number of active site. Thus as the concentration of the solution increases, more and more of these sites fill up until they are saturated. It has been demonstrated that when the concentration of the solution increases, the upward tendency of adsorption gradually reduces. At the same initial concentration, the removal rate and amount of Cr(VI) by GLC increased with ambient temperature, demonstrating that the adsorption process benefits from the increase in temperature. It's probable that when the temperature rose, the solution's viscosity decreased, and Cr(VI)’s ability to diffuse increased, improving GLC’s capacity to remove Cr(VI).

#### Adsorption isotherms

Three adsorption isotherm models were used to simulate the adsorption equilibrium curves of GLC at three temperatures. The fitting results are displayed in Fig. 6c. The detailed fitting parameters of the adsorption isotherms are listed in Table 2. It is clear that all three models are capable of accurately simulating the GLC adsorption data. The RP model, which suggests that the removal of Cr(VI) is the result of a mix of a chemical reaction and physical adsorption^71^, has the highest fitting R^2^ value among them. The Freundlich model's empirical constant 1/*n* ranges from 0.1 to 0.5, further supporting the idea that GLC samples chemisorb Cr(VI)^72,73^. The Langmuir model provided a good fit to the data, suggesting that chromium interacts with the GLC surface active site more on the monomolecular layer^52^. The Langmuir model's rate constant *K*_*1*_ values were in the range of 0–1 for all three temperatures, indicating that GLC possesses effective Cr(VI) adsorption. Using the Langmuir model, the maximum amounts of Cr(VI) by GLC were calculated to be 16.55 mg/g at 318 K, 24.13 mg/g at 308 K, and 29.55 mg/g at 298 K. With the rising temperature, the maximum adsorption amount increased, which is consistent with earlier research^52^. This shows that when the temperature rises, the adsorption capacity increases, which is good news for the adsorption process.

**Table 2:** Adsorption isotherm fitting parameters and thermodynamic parameters for Cr(VI) removal by GLC.

T(K)		298	308	318
Langmuir	Q_m_	16.55	24.13	29.55
	K_1_(L·mg^−1^)	0.11	0.12	0.14
	R^2^	0.9365	0.9938	0.9883
Freundlich	k_2_(mg^1–1/n^·L^1/n^·g^−1^)	5.34	6.21	7.43
	1/n	0.2	0.25	0.26
	R^2^	0.9054	0.9354	0.94
Redlich–Peterson	K_3_(L·g^–1^)	2.419	2.884	3.496
	α(L^β^·mg^−β^)	0.172	0.12	0.126
	β	0.98	1	0.984
	R^2^	0.9491	0.9999	0.9889
$\Delta {G}^{\theta }$	(KJ·mol)	− 11.9781	− 12.8302	− 13.7556
$\Delta {H}^{\theta }$	(KJ·mol)	14.4946		
$\Delta {S}^{\theta }$	(J·mol^−1^·K^−1^)	88.7935		

#### Adsorption thermodynamics

Thermodynamic analysis of the adsorption process can be used to ascertain the process's direction and driving force, as well as the effects of various parameters on adsorption. The calculation results of the thermodynamic parameter are listed in Table 2. It can be seen that the values of $\Delta {G}^{\theta }$ are less than 0 at the three temperatures, indicating that the adsorption of Cr(VI) by GLC proceeds spontaneously; the absolute values of $\Delta {G}^{\theta }$ rise as the ambient temperature rises, further demonstrating that the adsorption process benefits from higher temperatures. Furthermore, it suggests that the adsorption reaction is highly spontaneous and stable. The value of $\Delta {H}^{\theta }$ calculated by fitting is 14.494 kJ/mol, which is greater than zero, indicating that the adsorption process is a heat absorption reaction^74^. It is generally considered that the adsorption is mainly physical in the 0–20 kJ/mol range for $\Delta {H}^{\theta }$^75^. According to the computed value of $\Delta {S}^{\theta }$, which was 88.793 J/mol, the removal of Cr(VI) by GLC is a process that increases entropy and disorder^76^. During the adsorption process, the disordered state of Cr(VI) in the solution was adsorbed onto the surface of GLC. Then, it changed to an ordered state with reduced degrees of freedom. However, as the adsorption continues, the water molecules' degree of freedom on the GLC surface decreases^77^. The result is that the value of $\Delta {S}^{\theta }$ decreases.

### Adsorption mechanism

**Figure 7:**
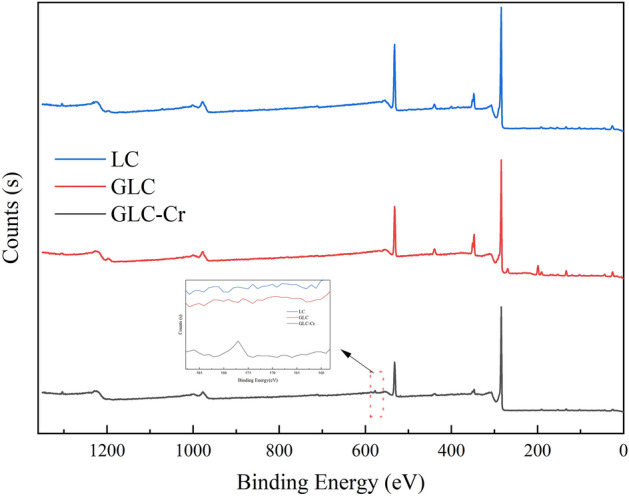
XPS full spectrum and partial magnification of the samples.

The sample surface element composition, chemical state, and molecular structure were revealed by the XPS spectrum's peak position and peak shape, while the sample surface element concentration was revealed by the peak intensity. The variations in their XPS spectra before and after adsorption (Fig. 7) were examined for comprehending how GLC absorbed Cr(VI). The ratio of the strength of the O 1 s and C 1 s spectral peaks is noticeably decreased after adsorption, and a distinct Cr 2p spectral peak can be seen on the material's surface.

**Figure 8:**
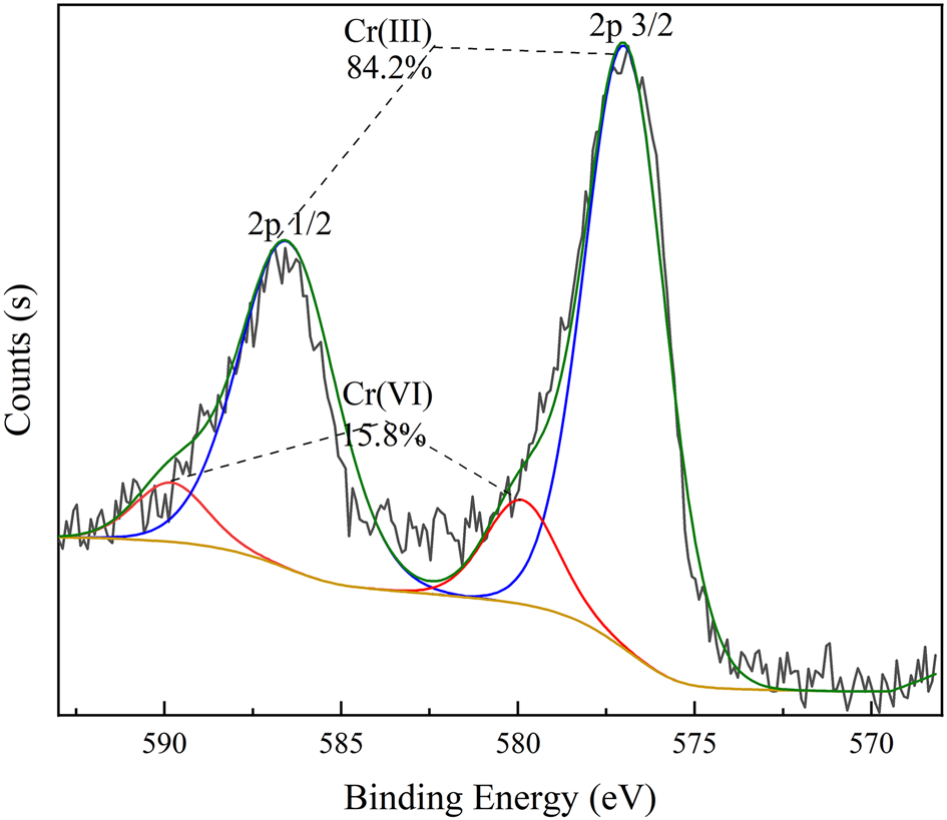
Split-peak fitted spectrogram of chromium.

It is inferred that Cr(VI) was successfully adsorbed on the material's surface and that the adsorption procedure altered the material's initial surface functional group structure. By fitting the Cr 2p spectral peaks after adsorption (Fig. 8), it was found that in addition to Cr(VI), a larger proportion of trivalent chromium was found on the GLC surface, with peaks located near 576 eV and 587 eV^30^, accounting for 84.2% of the Cr 2p spectral peaks. This indicates that most of the Cr(VI) adsorbed by GLC was converted to trivalent chromium and retained on the material surface, which is consistent with the results of the previous analysis. Many researchers also found that the redox reaction plays an important role in the removal of Cr(VI) in aqueous solutions^78^. Under acidic conditions, Cr(VI) can be reduced to Cr(III) by gaining electrons, and the possible reaction equations are as follows^79^:

16 $$\mathrm{HCr}{O}_{4}^{-}+7{H}^{+}+3{e}^{-}\to C{r}^{3+}+4{H}_{2}O,$$

17 $$2C{r}_{2}{O}_{7}^{2-}+14{H}^{+}+6{e}^{-}\to 2C{r}^{3+}+7{H}_{2}O.$$

**Figure 9:**
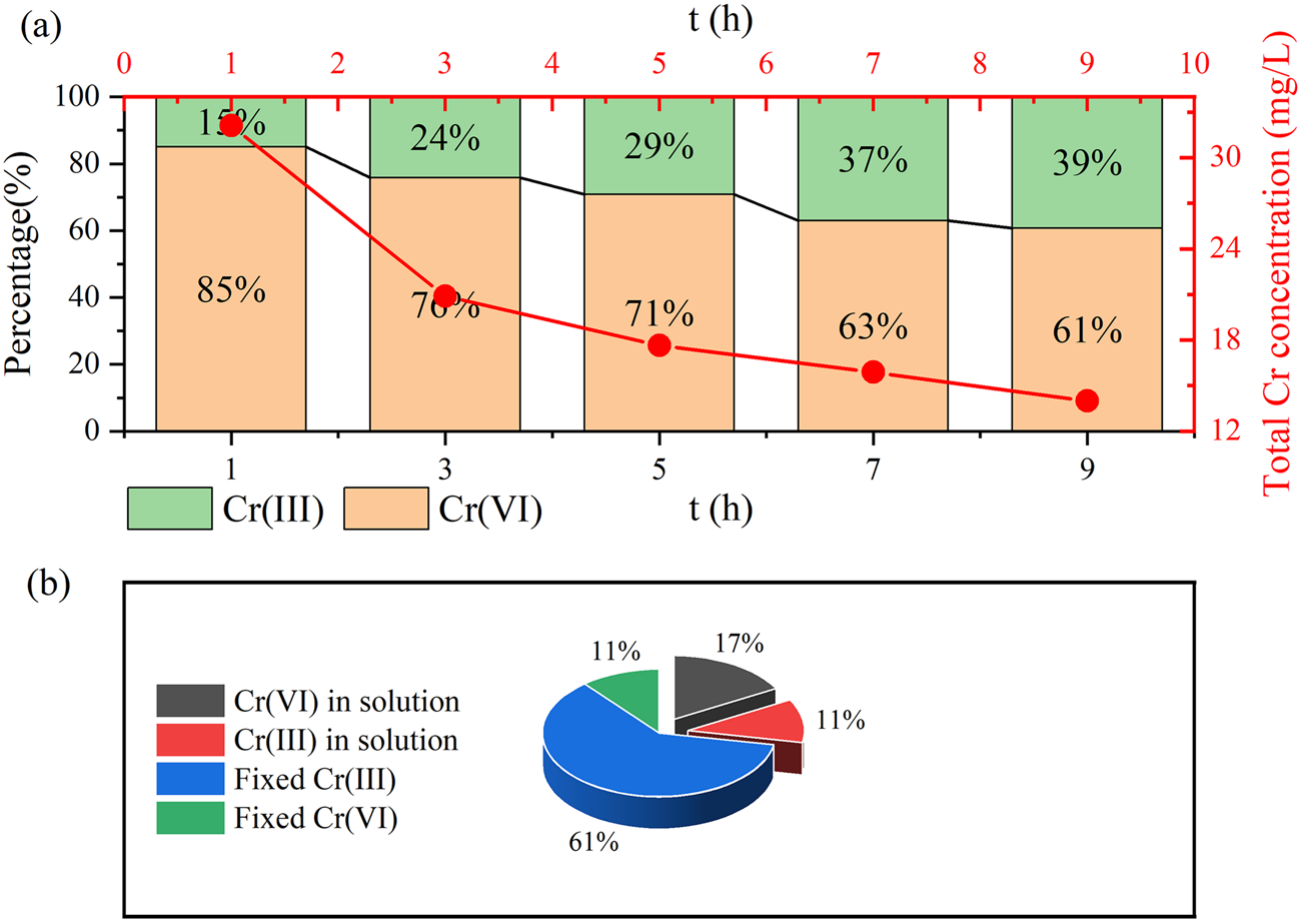
(**a**) Variations in the solution’s total concentration of chromium and the ratios of Cr(III) and Cr(VI) across different adsorption durations; (**b**) The percentage of different forms of chromium are displayed at adsorption equilibrium.

This further supports the idea that Cr(VI) elimination is facilitated by lower *pH*. Functional groups –OH and C=O can be used as electron donors for the reduction of Cr(VI)^80^. The –OH and C=O functional groups are more abundant in the potassium hydroxide-modified GLC than they are in the LC. Because of its considerable propensity to donate electrons, GLC performs better while removing Cr (VI). The results of the previous FTIR analysis also indicate that –OH and C=O functional groups were involved in the removal of Cr(VI). Therefore, the two key factors for reducing Cr (VI) are the free hydrogen provided by the solution and the electrons created by GLC. The created Cr(III) can adsorb on the GLC surface by complexation, substitution, and other processes and, of course, can be partially released into solution(in Fig. 9a)^81^. As the adsorption process continued, it was clear from Fig. 9a that while the concentration of total chromium in the solution was on the down, the concentration of Cr(III) in the solution was on the rise. It is inferred that as the amount of Cr(III) produced by reduction grew, more Cr(III) was released into the solution. We calculated the amount of Cr(VI) reduced to Cr(III) by the concentration of Cr(VI) and Cr(III) in the solution measured at the equilibrium of Cr(VI) adsorption under acidic conditions (*pH* 2), and then combined with the data obtained from XPS analysis. The results are shown in Fig. 9b to examine the contribution of reduction to Cr(VI) removal more visually. It demonstrates that at the adsorption equilibrium, Cr(VI) was converted to Cr(III) by 72% (11% + 61%). Even if the data acquired using XPS are not very exact, they are nevertheless sufficient to show the importance of reduction in the removal of Cr(VI).

Additionally, it is possible to electrostatically gravitate Cr(VI) anion in solution with the abundant oxygen-containing functional groups on the GLC surface, such as OH, C=O, and C–O, which are easily available as protons under acidic conditions^82^. To evaluate the contribution of each functional group to the removal of Cr(VI), the researchers calculated the binding energy between different functional groups and Cr_2_O_7_^–2^ and found that the binding energy values were − 6.74 eV for C–OH, – 5.55 eV for COOH, and − 2.72 eV for C^30^. It indicates that all these functional groups can be used as adsorption sites for Cr(VI). Cr(VI) may first bind to sites with low binding energy before moving on to sites with high binding energy^83^. Thus, Cr(VI) binds more readily to C–OH and less readily to C. Additionally, for OH functional group, if the background groups attached are different, the adsorption energy with Cr_2_O_7_^2–^ is also different^30^. This finding suggests that the background groups attached affect its ability to bind with Cr(VI).

**Figure 10:**
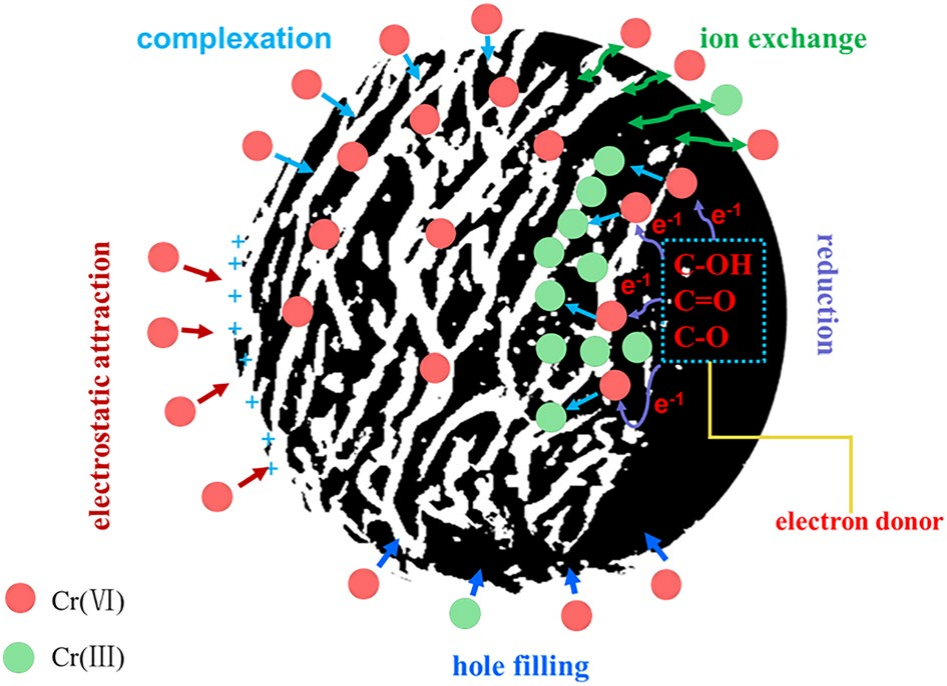
Diagram of the adsorption mechanism.

Based on the aforementioned numerous experimental findings, the reaction mechanism illustrating the process of Cr(VI) adsorption onto GLC has been anticipated and is depicted in Fig. 10. The adsorption mechanism of Cr(VI) includes physical adsorption and chemisorption. Physical adsorption mainly involves electrostatic attraction and pore filling, while chemisorption mainly involves redox and functional group complexation^30^. In the adsorption of Cr(VI) by GLC, these mechanisms do not exist independently. Cr(VI) fixation may require the use of multiple mechanisms. Cr(VI) in solution may first be attracted to the pore surface of the material by electrostatic interactions. Then a chemical bond with the surface functional group was formed. During this process, the electrons of the surface functional group were transferred to Cr(VI), which was reduced to Cr(III) and remained on the surface of the material. Part of the formed Cr(III) may release into the solution due to the low bonding ability of Cr(III) with the original group as well as electrostatic repulsion. Part of the Cr(III) ions can also be freshly bound to the GLC surface by ion exchange or complexation.

## Conclusion

Blue coke is a product of low-temperature dry distillation of coal. It contains a certain proportion of pores and has a certain heavy metal adsorption capacity. By the modification of potassium hydroxide, the ash originally filled in the pores was removed, and many new pores were generated. As a result, GLC has a more porous structure. The specific surface area of GLC was three times more than that of LC, with an average pore size of 2.917 nm. The modification altered the functional structure of the material surface, leading to a considerable increase in the amount of hydroxyl functional groups. Additionally, it also damaged the graphitic microcrystalline structure of the material, which had a tendency to transition into amorphous carbon. The maximum adsorption capacity fitted by Langmuir at room temperature was 16.55 mg/g. The adsorption of Cr(VI) by GLC is a spontaneous, heat-absorbing, and entropy-increasing process. Therefore, increasing the temperature is beneficial to the removal of Cr(VI) by GLC. The intermittent adsorption experiments showed that Cr(VI) was both physisorbed and chemisorbed on the GLC surface. Pore filling, electrostatic attraction, redox, and complexation reactions all took place during the adsorption of Cr(VI).

## Data Availability

All data generated or analysed during this study are included in this published article.

## Abbreviations

Cr(VI): Hexavalent chromium
LC: Blue coke powder
GLC: Blue coke powder modified by KOH
XRD: X-ray diffraction
XPS: X-ray photoelectron spectroscopy
FE-SEM: Field emission scanning electron microscope
FTIR: Fourier transform infrared radiation
R: Removal rate
PFO: Pseudo-first-order
PSO: Pseudo-second-order
WM: Weber-Morris
pHpzc: The point of zero electric charges
RP: Redlich-Peterson
IUPAC: International union of pure and applied chemistry
